# Deficiency of immunity to poliovirus type 3: a lurking danger?

**DOI:** 10.1186/1471-2334-12-24

**Published:** 2012-01-26

**Authors:** Claudia Reinheimer, Imke Friedrichs, Holger F Rabenau, Hans W Doerr

**Affiliations:** 1 Institute for Medical Virology, University Hospital Frankfurt am Main, Paul Ehrlich-Straße 40, 60596 Frankfurt am Main, Germany

**Keywords:** Poliomyelitis, Vaccination, Seroepidemiology, Lack of immunity, Germany

## Abstract

**Background:**

Europe was certified to be polio-free in 2002 by the WHO. However, wild polioviruses remain endemic in India, Pakistan, Afghanistan, and Nigeria, occasionally causing polio outbreaks, as in Tajikistan in 2010. Therefore, effective surveillance measures and vaccination campaigns remain important. To determine the poliovirus immune status of a German study population, we retrospectively evaluated the seroprevalence of neutralizing antibodies (NA) to the poliovirus types 1, 2 and 3 (PV1, 2, 3) in serum samples collected from 1,632 patients admitted the University Hospital of Frankfurt am Main, Germany, in 2001, 2005 and 2010.

**Methods:**

Testing was done by using a standardized microneutralization assay.

**Results:**

Level of immunity to PV1 ranged between 84.2% (95%CI: 80.3-87.5), 90.4% (88.3-92.3) and 87.5% (85.4-88.8) in 2001, 2005 and 2010. For PV2, we found 90.8% (87.5-90.6), 91.3% (89.3-93.1) and 89.8% (88.7-90.9), in the same period. Seroprevalence to PV3 was 76.6% (72.2-80.6), 69.8% (66.6-72.8) and 72.9% (67.8-77.5) in 2001 and 2005 and 2010, respectively. In 2005 and 2010 significant lower levels of immunity to PV3 in comparison to PV1 and 2 were observed. Since 2001, immunity to PV3 is gradually, but not significantly decreasing.

**Conclusion:**

Immunity to PV3 is insufficient in our cohort. Due to increasing globalization and worldwide tourism, the danger of polio-outbreaks is not averted - even not in developed countries, such as Germany. Therefore, vaccination remains necessary.

## Background

Poliovirus, the etiologic agent of paralytic poliomyelitis (hereinafter referred to as polio), is a positive-sensed single-stranded RNA virus, which belongs to the genus *Enterovirus*. Enteroviruses in turn are a species of small, pathogenic, icosahedral viruses belonging to the family *Picornaviridae*. Polioviruses are subdivided in three immunologically different serotypes: poliovirus type 1, 2 and 3 (hereinafter referred to as PV1, 2, 3). Since 1960, polio has been controlled by the use of live oral polio vaccine (OPV) or inactivated polio vaccine (IPV). The latter poses no risk of vaccine-associated paralytic polio and has been used in Germany since 1998 [1,2]. Polio usually affects children under the age of 5 years: paralytic polio has been seen in one of 200 cases, fatal cases are seen in about 5-10% of paralytic polio in developing countries [3]. The number of polio cases has been reduced by > 99%, from an estimated number of 350,000 cases worldwide in 1988 [4] to 1,606 cases in 2009 [4] and 1,294 cases in 2010 [5]. While Germany was deemed to be polio-free by WHO on June, 21^st ^in 2002 [6], circulating wild polioviruses remain endemic in four major locations: Nigeria, Pakistan, Afghanistan and India [4,7,8]. The occurrence of polio outbreaks, such those reported in Tajikistan, Turkmenistan and Kazakhstan in 2010 [9,10] or Angola in 2007/2008 [11] underlines the necessity of vaccination campaigns for polio prevention. Epidemiological surveillance is also crucial to document polio-absence in polio-free countries. Between 1997 and 2010 Germany has taken part in the acute flaccid paralysis (AFP)-program, which was initiated to exclude a PV-infection by a two-time stool investigation in cases of AFP in children younger than 15 years. Because of low participation rates an alternative surveillance-program was initiated in Germany by the *National Commission for Polio-Eradication in the Federal Republic of Germany *(*Nationale Kommission für die Polioeradikation in der Bundesrepublik Deutschland*) and the Robert Koch-Institut (RKI), Berlin, Germany, in 2005. It is the so called *Enterovirus-Surveillance*, which bases on laboratory diagnostic clarification of viral meningitis or encephalitis by stool or cerebrospinal fluid (CSF). By *Enterovirus-Surveillance *Germany fulfils the obligation to prove to be and remain polio-free, as required by WHO. Up to date, no polio-caused AFP case has been detected in Germany [12]. The objective of our study was to describe the immunity status to PV1, 2, 3 of a German urban cohort 13 years after introduction of IPV and 9 years after elimination of polio in Germany. Therefore, we retrospectively evaluated the seroprevalence of neutralizing antibodies (NA) to PV1, 2, 3 using microneutralization assay (MNA) in 1,632 routinely collected serum samples in the university-hospital of Frankfurt am Main, Germany. Furthermore, we quantitatively analyzed and evaluated the level of immunity to PV1, 2, 3 by titres on average.

## Methods

In the years 2001, 2005 and 2010 we tested 411, 878 and 343 serum samples, respectively, for NA to PV1, 2, 3. The serum samples were obtained from patients admitted to the University Hospital of Frankfurt am Main, Germany (FRA).

As we conducted an unlinked anonymous retrospective study, patients' data regarding age and sex were available. However, data concerning nationality, residence or concomitant diseases were not available.

The samples were routinely screened for NA against PV1, 2, 3 by MNA. MNA and controls are performed by an in-house procedure according to WHO guidelines [13] using vaccine-PV-strains. Currently, trivalent IPVs are mainly produced using the poliovirus strains Mahoney (PV1), MEF-1 (PV2), and Saukett (PV3), grown in Vero cell line [14,15] The strains were obtained from the German reference laboratory for poliomyelitis and enteroviruses at the Robert Koch Institut, Berlin. The MNA was performed as previously described [16]. Briefly: Sera were inactivated at 56°C for 30 min before use, diluted two-fold from 1:10 to 1:1280 and then incubated for 1 h at 37°C (in a CO_2 _incubator) with 100 tissue culture infective dose_50 _(TCID_50_) of challenge virus (either PV1, 2 or 3). After the incubation period 50 μl of the serum-virus suspension was added to 50 μl of Vero cells suspension (African green monkey kidney, ATCC CCL-81). Cell controls and a reference serum of known PV1,2,3 neutralizing activity was included in each test to examine reproducibility of results. Each test serum was investigated in triplicate. After incubation for 5-7 days, the highest dilution of serum that prevents the development of virus induced cytopathogenic effects (CPE) was recorded. The NA titre corresponded to the reciprocal of this dilution. A serum sample was considered positive, if antibodies were present at a dilution 1: ≥ 10 of the serum specimen.

Statistical analysis was done by using the 95% confidence interval (95%CI) with a significance level of *p *< 0.05, Chi-Square test or the Fisher's exact test in the case of low sample numbers by using the program BIAS for Windows 8.3 (Epsilon Verlag, Hochheim Darmstadt 2007). Differences were regarded significant with an error probability of *p *< 0.05. The Kruskal-Wallis test was used for analyzing the association of seroprevalence and age. Results are presented in modification of age groups defined by WHO. Modification in this context means consolidation of 2 or 3 age groups within a single because of low sample numbers in the age group recommended by WHO. We furthermore used three, previously defined [16] NA titre ranges and used them to quantify the level of immunity to PV1, 2, 3. Thus, low, medium and high immunity is given for titre ranges between 1:10-1:20 and 1:40-1:160 and 1:320-1:1280, respectively.

## Results

**Figure 1:**
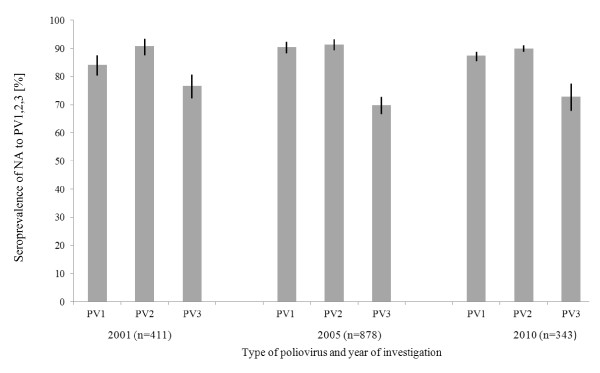
**Seroprevalence of NA to PV1, PV2, PV3 in the years 2001, 2005 and 2010 (95%CIs are represented by black lines)**.

A non-age-specific, overall evaluation of PV1, 2, 3-NA is shown in Figure 1. Seroprevalence (with 95%CI in brackets) of NA to PV1 amounts 84.2% (80.3-87.5), 90.4% (88.3-92.3) and 87.5% (85.4-88.8) in 2001, 2005 and 2010, respectively. Highest levels of seropositivity were detectable for PV2 with 90.8% (87.5-90.6), 91.3% (89.3-93.1) and 89.8% (88.7-90.9) in 2001, 2005 and 2010, respectively. Lowest seroprevalences were observed for PV3: values ranged between 76.6% (72.2-80.6), 69.8% (66.6-72.8) and 72.9% (67.8-77.5) in 2001 and 2005 and 2010, respectively.

**Figure 2:**
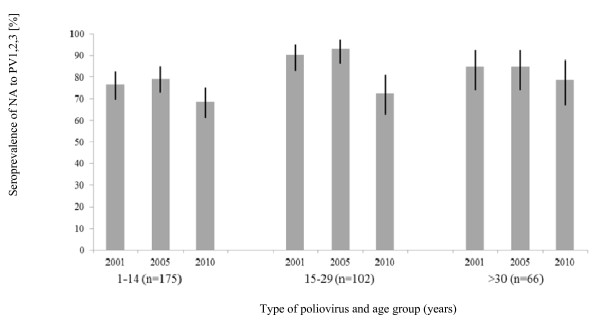
**Age-specific seroprevalence of NA to PV1, PV2, PV3 in 2010 (95%CIs are represented by black lines)**.

In Figure 2 the age-specific seroprevalence of NA to PV1, 2, 3 is illustrated. In the age group of 15-29 years highest level of seropositivity to PV1 (all values in [%]: 90.2; 95%CI: 82.7-95.2) and PV2 (93.1; 86.3-97.2) were recorded. Highest level of seroprevalence to PV3 (78.8; 66.9-87.8) was detected in the group aged > 30 years. In comparison to PV1 and PV2, all the age groups show a lower level of seroprevalence of anti-PV3. In the group aged 15-29 years a significant lower seroprevalence to PV3 (72.5; 62.8-80.9) than to PV1 (90.2; 82.7-95.2) and PV2 (93.1; 86.3-97.2) was detectable (p < 0.05).

**Figure 3:**
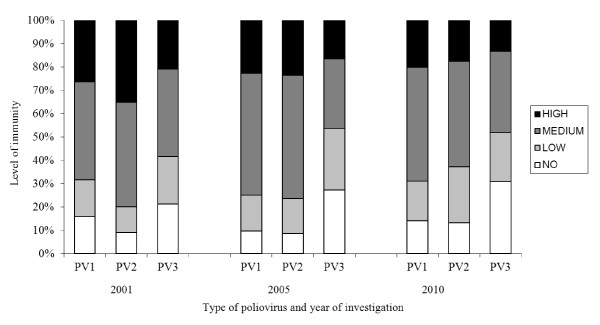
**Immunity to PV1,2,3: titre-classes in 2001, 2005 and 201**. Immunity levels: HIGH = 1: > 320; MEDIUM = 1:40-1:160; LOW = 1:10-1:20; NO = < 1:10.

**Figure 4:**
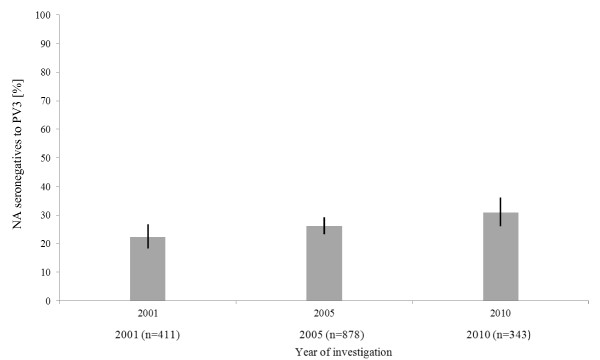
**Lack of immunity to PV3 (95% CI are represented by black lines)**.

A qualitative analysis of the NA titres using three levels of immunity (low [1:10-1:20], medium [1:40-1:160], high [1: > 320]) was used to assess the level of immunity to PV1, 2, 3. No immunity is given for titres lower than 1:10. Figure 3 illustrates the distribution to these immunity-levels. It shows a decreasing number of PV3-"high immunity"-sera from 2001 to 2005 and 2010 while the percentage of samples with "no immunity" is significantly increased over years (*p *= 0.008; see Figure 4).

## Discussion

From the epidemiological point of view, eradication of polio is a matter of urgency. The spread of wild polioviruses from endemic areas to polio-free countries remains a potential risk, as vaccination coverage rates can decrease, and vaccine-induced immunity can wane. For this reasons, vaccination campaigns and epidemiological surveillance are absolutely necessary to maintain and verify polio-absence in polio-free countries.

In our study population, we found a distinctive gap for PV3. This gap has previously been described by Weber et al. [16], Wicker et al. [17], Diedrich et al. in [18,19], Franck et al. [20], but we found a further increase of this hiatus. In fact, also in our study the immunity level to PV3 is the lowest among all types of poliovirus [16-26]. This result can be explained by a reduced antigenicity immunogenicity of PV3 when compared to PV1 and PV2 [26]. This should be an aspect of interest in future vaccine development. An interesting aspect is that lowest seroprevalences of antibodies to PV1 - 3 were observed in the group aged 1-14 years (Figure 2). This phenomenon may be coherent with the implementation of IPV in Germany in 1998 [1,2]. When OPV was used, no such gap was observed [21].

Not only from this point of view but also in consideration of globalisation and worldwide tourism, polio remains an important theme: due to travel by air, importation of PV from endemic areas can occur within hours by air traffic. In Germany, 10-20% of inhabitants are immigrants [27,28], and may frequently invite or visit their relatives abroad. Consequently the risk of PV-import to Germany from endemic countries is present, and necessitates maintenance of an immune population until polio is eradicated globally.

Available data on immunological status to PV from other countries should be compared with caution, since determination of neutralizing antibodies is a biological assay with big deviations [28].

In Portugal 91.6%, 94.2% and 75% of the tested persons had shown protective antibodies to PV 1,2,3, respectively, in 2002 [22]. Immigrants from less developed countries in North-East Italy were seropositive to PV 1, 2, 3 in 98.3%, 99.6% and 95.9%, respectively [23]. Furthermore in Israel, 18 year-old soldiers recruited to the Israel Defence Forces tested for antibodies to PV1, 2, 3 were found to be protected in 98.7%, 99.6% and 96.4%, respectively [24]. In Jilin Region, China, 95%, 94.6% and 92.3%, had positive test results of NA against Polio type 1,2,3, respectively [25]. In comparison to our PV-seroprevalences (and also to former studies from Germany [16,18-21]) the results mentioned above are markedly higher. The reason for this phenomenon is unclear. In which way it is influenced by the way of titration, definition of the cut-off titre and counting of dilution (final dilution vs. serum dilution) remains unclear.

In a population of women attending antenatal in southern India, nearly 50% had shown no immunity (titres 1: < 8) against polio, with the lowest levels for PV3 and 1 [29]. Nepal, a country three of four endemic countries, also shows hard effort in the fight against polio. However, while high acceptance of vaccination-programs is documented [30], seroprevalence data are lacking. Seroprevalence surveys are necessary to identify population-categories at risk to be susceptible for polio, and to target specific vaccination activities to protect these groups. The availability of PV 1 and PV 3 monovalent vaccines vaccine could provide the global poliomyelitis eradication initiative with additional vaccine options [31], even though it could complicate decision making on vaccination policies.

Optimization of immunity to PV3 is a matter of urgency. As mentioned above and shown in Figure 1,2,3,4, seroprevalence of NA to PV3 has been decreasing in Germany for several years. Therefore, continuing vaccination campaigns are as important as monitoring the population's immunity to sustain the present polio-free situation.

## Conclusions

Despite intensive efforts in the fight against polio, it remains endemic in India, Pakistan, Afghanistan and Nigeria. Therefore, vaccination campaigns are as important as maintaining surveillance to monitor enteroviral events - including those countries, which are deemed to be polio-free by WHO. Even if the European countries are known to be polio-free the danger has not been completely averted: we observed a distinctive gap in immunity to PV3 in all age-groups and furthermore a decreasing immunity to PV, overall. Thus, we learn that vaccination against polio is absolutely necessary further on to preserve our present polio-free situation.

## Abbreviations

95%CI: 95% confidence interval; AFP: acute flaccid paralysis; CPE: cytopathogenic effect; CSF: cerebrospinal fluid; IPV: inactivated polio vaccine; MNA: microneutralization assay; NA: neutralizing antibodies; OPV: live oral polio vaccine; PV: Poliovirus; PV1: 2: 3: Poliovirus type 1: 2: 3; RKI: Robert Koch-Institut: Berlin: Germany; WHO: World Health Organisation.

## Competing interests

The authors declare that they have no competing interests.

## Authors' contributions

*CR *coordinated data collection, wrote first draft and revisions of the paper, corresponding author; *IF *reviewed drafts of paper; *HFR *assisted with data collection, reviewed drafts of paper. *HWD *assisted with data analysis and reviewed drafts of paper; chief investigator of the study. All co-authors read and approved the final manuscript.

## Pre-publication history

The pre-publication history for this paper can be accessed here:

http://www.biomedcentral.com/1471-2334/12/24/prepub
